# Targeting pain and inflammation by peripherally acting opioids

**DOI:** 10.3389/fphar.2013.00123

**Published:** 2013-09-23

**Authors:** Christoph Stein

**Affiliations:** Department of Anesthesiology and Critical Care Medicine, Charité Campus Benjamin Franklin Freie Universitaet BerlinBerlin, Germany

**Keywords:** opioid receptor, opioid peptide, inflammation, pain, analgesia

## Introduction

Opioids can produce potent analgesia by activating opioid receptors outside the central nervous system, thus avoiding centrally mediated unwanted effects. Peripheral opioid receptors are expressed in peripheral sensory (dorsal root ganglion) neurons and can interact with exogenous or endogenous opioid ligands both in animals and in humans. Inflammation of peripheral tissue leads to upregulation of such opioid receptors and to local production of endogenous opioid peptides in immune cells. This article will summarize recent mechanistic, preclinical, and clinical findings.

## Opioid receptor signaling in peripheral sensory neurons

Co-localization and electrophysiological studies have confirmed the presence of opioid receptors on C- and A-fibers, on dorsal root ganglion neurons expressing transient receptor potential vanilloid subtype-1 (TRPV-1) and G-protein-coupled inwardly rectifying K^+^ (GIRK) channels, and on fibers expressing isolectin B4, substance P, and/or calcitonin-gene-related peptide, consistent with the phenotype of nociceptors. The activation of such opioid receptors results in inhibition of high-voltage activated Ca^++^- and enhancement of GIRK-currents. These effects are mediated by G-proteins (G_i_ and/or G_o_). In addition, opioids—via inhibition of adenylyl cyclase—suppress tetrodotoxin-resistant Na^+^-, TRPV1- and other non-selective cation currents stimulated by inflammatory agents, which may account for the notable efficacy of peripheral opioids in inflammatory and neuropathic pain. Consistent with their effects on ion channels, opioids attenuate the excitability of peripheral nociceptor terminals, the propagation of action potentials, the release of excitatory proinflammatory neuropeptides (substance P, calcitonin gene-related peptide) from peripheral sensory nerve endings, and vasodilatation evoked by stimulation of C-fibers. These mechanisms result in analgesia and/or anti-inflammatory actions (Endres-Becker et al., 2007; Vetter et al., 2008; Stein and Machelska, 2011; Moshourab and Stein, 2012; Nockemann et al., 2013; Spahn et al., 2013; Stein and Küchler, 2013).

## Peripheral opioid receptors and tissue injury

Peripheral opioid analgesic effects are particularly prominent in inflamed tissue (Kalso et al., 2002; Stein et al., 2003; Vadivelu et al., 2011). Under such conditions the synthesis and expression of opioid receptors in dorsal root ganglia is elevated. Subsequently, the axonal transport and membrane-directed trafficking of opioid receptors increases, leading to their upregulation on peripheral neuron terminals (Patwardhan et al., 2005; Cayla et al., 2012; Pettinger et al., 2013). These events are dependent on neuronal electrical activity, cytokines, and nerve growth factor from the damaged tissue. In mechanical nerve injury leading to neuropathic pain, opioid receptors accumulate proximal and distal to the lesion, indicating anterograde and retrograde transport (Labuz et al., 2009). Inflammatory milieu (low pH, prostanoids, bradykinin) can augment opioid receptor function e.g., by more efficient G-protein coupling and inhibition of elevated neuronal cyclic adenosine monophosphate production (Stein and Machelska, 2011; Stein, 2013). Inflammation also leads to sprouting of sensory nerve terminals and disruption of the perineurial barrier, thus facilitating the access of opioid agonists to their receptors (Rittner et al., 2012). Endogenous opioid ligands derived from inflammatory cells stimulate recycling of opioid receptors to the membrane of sensory neurons, which can prevent the development of tolerance to peripherally active opioid agonists (Zöllner et al., 2008). Consistently, clinical studies have indicated a lack of cross-tolerance between peripheral exogenous and endogenous opioids in synovial inflammation. All of these mechanisms can contribute to enhanced antinociceptive efficacy of opioid agonists in injured tissue (Stein and Machelska, 2011).

## Endogenous ligands of peripheral opioid receptors

Concurrent with the development of inflammation, opioid peptide-producing immune cells are recruited to the site of injury. The most thoroughly characterized peptides are β-endorphin and enkephalins deriving from the respective precursors proopiomelanocortin (POMC) and proenkephalin. Transcripts and peptides derived from POMC and proenkephalin, as well as the prohormone convertases PC1/3 and PC2, necessary for their posttranslational processing, were detected in such cells. The expression of immune-derived opioids is stimulated by viruses, endotoxins, cytokines, corticotropin releasing hormone (CRH) and adrenergic agonists. In painful tissue inflammation and neuropathy, POMC mRNA, β-endorphin, met-enkephalin, and dynorphin are detectable in circulating cells and lymph nodes, and are upregulated in resident lymphocytes, monocytes/macrophages, and granulocytes. Circulating opioid-containing leukocytes migrate to injured tissue attracted by adhesion molecules, chemokines, and neurokinins. In inflamed tissue, opioid-containing leukocytes, vascular P-selectin, ICAM-1, and PECAM-1 are simultaneously upregulated. Blocking chemokines, selectins, or ICAM-1 reduces the extravasation of opioid-containing cells and increases inflammatory and neuropathic pain. Consistently, immunosuppression can exacerbate pain (Labuz et al., 2009; Stein and Machelska, 2011; Busch-Dienstfertig et al., 2012).

Stimuli such as environmental stress, noradrenaline, CRH, interleukin-1β, chemokines, or mycobacteria can elicit opioid peptide release from immune cells via specific receptors and the regulated secretory pathway. Depending on the cell type and agent, intracellular Ca^++^ release from endoplasmic reticulum or extracellular Ca^++^ is required. *In vivo*, the secreted opioid peptides bind to opioid receptors on sensory neurons and elicit analgesia in injured tissue and neuropathy (Labuz et al., 2009; Rittner et al., 2009). Not only stimulated but also tonic release of opioids from immune cells decreases pain in animals (Rittner et al., 2009) and in humans (Stein et al., 1993). Thus, the development of inflammatory and neuropathic pain is counteracted by immune cells producing and secreting opioid peptides. Gene therapeutic approaches are aiming to increase the production of opioid peptides and receptors in inflammatory cells and peripheral sensory neurons, respectively (Stein and Machelska, 2011; Raja, 2012). Preventing the extracellular degradation of endogenous opioid peptides by peptidase inhibitors as well as nanocarrier-directed transport of opioids have been shown to diminish inflammatory pain (Roques et al., 2012; Schreiter et al., 2012; Hua and Cabot, 2013).

## Preclinical studies on peripheral opioid analgesics

This basic research has stimulated the development of novel opioid ligands acting exclusively in the periphery without central side-effects. A common approach is the use of hydrophilic compounds with minimal capability to cross the blood-brain-barrier. Among the first compounds were the mu-agonist loperamide (known as an antidiarrheal drug) and the kappa-agonist asimadoline. Peripheral restriction was also achieved with glucuronidation, arylacetamide (ADL 10-0101), morphinan-based (TRK-820, HS-731), triazaspiro (DiPOA) and peptidic compounds (DALDA, FE200665, CR845). While earlier attempts to demonstrate peripheral opioid analgesia in normal tissue failed, they were much more successful in models of pathological pain (Stein, 1993). For example, in subcutaneous inflammation the local injection of low, systemically inactive doses of mu-, delta-, and kappa-agonists produces dose-dependent and opioid receptor-specific antinociception. Such effects were also shown in models of nerve damage, visceral, thermal, cancer and bone pain (Stein and Machelska, 2011).

## Effects on inflammation

Inflammation contributes to many diverse disorders such as trauma, arthritis, neuropathy, fibromyalgia, endometriosis, diabetes, cancer, and chronic pain. Therapeutic inhibition of inflammation is indicated when it becomes dysregulated, chronic, recurrent or inappropriate. However, standard treatments such as steroids, non-steroidal anti-inflammatory drugs (NSAIDs), and disease-modifying drugs have severe side effects (ulcers, bleeding, myocardial infarction, stroke, infections) (Trelle et al., 2011) and biological anti-inflammatory treatments such as inhibitors of tumor necrosis factor-α or of Janus kinases can only be used in a limited number of patients due to their prohibitive cost, parenteral formulation and risk for infection and tumor induction. A large number of *in vitro* and animal investigations have produced evidence that peripherally active opioids can reduce release of proinflammatory neuropeptides, cytokines, plasma extravasation, vasodilation, immune mediators, expression of adhesion molecules and tissue destruction (Stein and Küchler, 2012). In contrast to currently available anti-inflammatory agents, opioids have no demonstrated organ toxicity, making them interesting candidates for drug development. However, there is a lack of clinical studies in this area at present.

## Clinical studies on peripheral opioid analgesics

The most extensively examined clinical application is the intraarticular injection of morphine. Both in human and veterinary medicine, numerous controlled clinical studies have demonstrated dose-dependent and peripherally mediated reduction of pain and/or supplemental analgesic consumption without significant side effects (Kalso et al., 2002; Stein, 2013). Intraarticular morphine is effective in acute (postoperative) and chronic (arthritic) pain, its effect is similar to intraarticular local anesthetics and steroids, and it is long lasting, possibly due to anti-inflammatory activity. Locally applied opioids were also effective in dental pain, skin ulcers, corneal abrasions and visceral pain (Sawynok, 2003; Farley, 2011; Vadivelu et al., 2011). Some studies found no peripheral effects of opioids, e.g., after injection into the non-inflamed environment along nerve trunks (Picard et al., 1997). The latter observation suggests that intraaxonal opioid receptors are “in transit,” and not available as functional receptors at the membrane. Peripherally restricted opioids are under investigation for human use (morphine-6-glucuronide, CR845), and were shown to reduce postoperative and visceral pain with similar efficacy as morphine but limited central side-effects (Dahan et al., 2008; Binning et al., 2011; Stein and Machelska, 2011).

## Summary

Opioids can reduce pain and inflammation by activating opioid receptors outside the central nervous system. Inflammation of peripheral tissue leads to upregulation of opioid receptors on peripheral sensory neurons and to local production of endogenous opioid peptides in immune cells. Future aims in drug development include the design of peripherally restricted opioid agonists, selective targeting of opioids to sites of painful injury and the augmentation of peripheral ligand and receptor synthesis, e.g., by gene therapy. The ultimate goal is to avoid detrimental side effects of currently available opioid and nonopioid drugs such as apnoea, cognitive impairment, addiction, gastrointestinal bleeding, and thromboembolic complications.
